# Fully covered self‐expandable metal stent placed over a stapled colon anastomosis in an animal model: A pilot study of colon metabolism over the stent

**DOI:** 10.1002/jgh3.12747

**Published:** 2022-05-06

**Authors:** Ioannis Oikonomakis, Daniel T Jansson, Per Skoog, Kristofer F Nilsson, Adrian D Meehan, Tal M Hörer, Kjell Jansson

**Affiliations:** ^1^ Department of Surgery, Colorectal Unit, Faculty of Medicine and Health Örebro University Örebro Sweden; ^2^ Medical University of Gdansk Gdansk Poland; ^3^ Department of Vascular Surgery and Institute of Medicine, Department of Molecular and Clinical Medicine Sahlgrenska University Hospital and Academy Gothenburg Sweden; ^4^ Örebro University and Sahlgrenska University, Faculty of Medicine and Health Örebro Sweden; ^5^ Department of Cardiothoracic and Vascular Surgery, Faculty of Medicine and Health Örebro University Örebro Sweden; ^6^ Department of Geriatrics, Faculty of Medicine and Health Örebro University Örebro Sweden

**Keywords:** basic experimental study, clinical practice and treatment, colorectal cancer, endoscopy, gastroenterology

## Abstract

**Background and Aim:**

Anastomotic leakage (AL) in colorectal resection and primary anastomosis is a common and feared complication. Fully covered self‐expandable metal stents (FCSEMSs) have been used for the treatment of AL. It is still unknown whether FCSEMSs affect anastomosis healing negatively by causing ischemia. In an animal study, we investigated the metabolic effects over a FCSEMS covering a stapled colon anastomosis.

**Methods:**

Seven pigs were investigated using microdialysis after laparotomy, colon resection, and anastomosis with stent placement. Measurements were done at the proximal and distal ends of the anastomosis and at a reference catheter placed at the small intestine. Measurements of glucose, pyruvate, lactate, glycerol, and the lactate/pyruvate ratio (L/P) were carried out.

**Results:**

Lactate and L/P were significantly higher at the oral part of the anastomosis, while glucose showed a small declining tendency. At the distal part of the anastomosis, glucose decreased significantly after the resection but did not reach zero. Lactate increased significantly whereas L/P increased only slightly. Glycerol levels were stable.

**Conclusion:**

Colon resection caused initially hypermetabolism in the intestinal ends next to the resection site. This hypermetabolism neither deteriorated nor turned into ischemia during the initial postoperative course, but the start of hypoxemia could not be excluded during the study and after the placement of an FCSEMS.

## Graphical abstract

Fully covered self‐expandable metal stents (FCSEMS) have been used for treatment of anastomotic leakage. It is still unknown if FCSEMS affect anastomosis healing negatively by causing ischemia. In our study, the colon resection and stent insertion caused hypermetabolism but not ischemia in the intestinal ends next to the resection site.

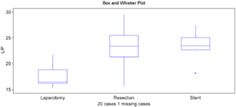

## Introduction

Anastomotic leakage (AL) after colorectal surgery is a common and dreaded complication. The incidence of AL after colon cancer surgery in Sweden in 2019 was reported by the Regional Cancer Center to be 4.1%. Thirty‐day mortality after acute operation is 6.1% and after elective surgery 1.4%.^1^ AL in colorectal surgery is usually treated with reoperation, or conservatively by fasting, antibiotics, and draining of abscesses.^2^, ^3^

Intraluminal covering stents are being used as a nonoperative alternative for the treatment of benign and malignant strictures in the gastrointestinal tract.^4^, ^5^ Very recently, covered intraluminal stents have been successfully introduced to manage AL after oesophagectomy, gastric bypass, and gastric sleeve operations.^6^, ^7^, ^8^ However, there are only a few clinical case reports and an animal study that describe the use of covered stents in colorectal AL.^9^, ^10^, ^11^, ^12^

Intraperitoneal microdialysis (IPM) has been introduced as a promising method for the prediction of surgical complications after gastrointestinal surgery.^13^, ^14^, ^15^, ^16^, ^17^, ^18^, ^19^ IPM studies have been conducted in both animals and humans.^20^, ^21^, ^22^ An increase of the intraperitoneal (IP) lactate/pyruvate ratio (L/P) is indicative of an increased anaerobic metabolism that may develop into inflammation, splanchnic hypoxia, and ischemia.^13^, ^14^, ^15^, ^16^, ^17^, ^18^, ^19^ This results in a disturbance of the anastomosis healing. These early metabolic changes are detectable before several postoperative complications such as AL and abdominal compartment syndrome.^23^ Biochemical measurements have been made and analyzed in subcutaneous and IP locations.^24^ Changes are observed only in the IP measurements before complications, suggesting that major surgical complications are preceded by splanchnic hypoxia/ischemia and that the changes are possible to be measured by IPM.^13^, ^14^, ^15^, ^16^, ^17^, ^18^, ^19^, ^25^, ^26^, ^27^, ^28^ Furthermore, it has been shown that obese and diabetic patients do not differ in postoperative IP L/P compared with controls.^24^, ^29^

**Figure 1:**
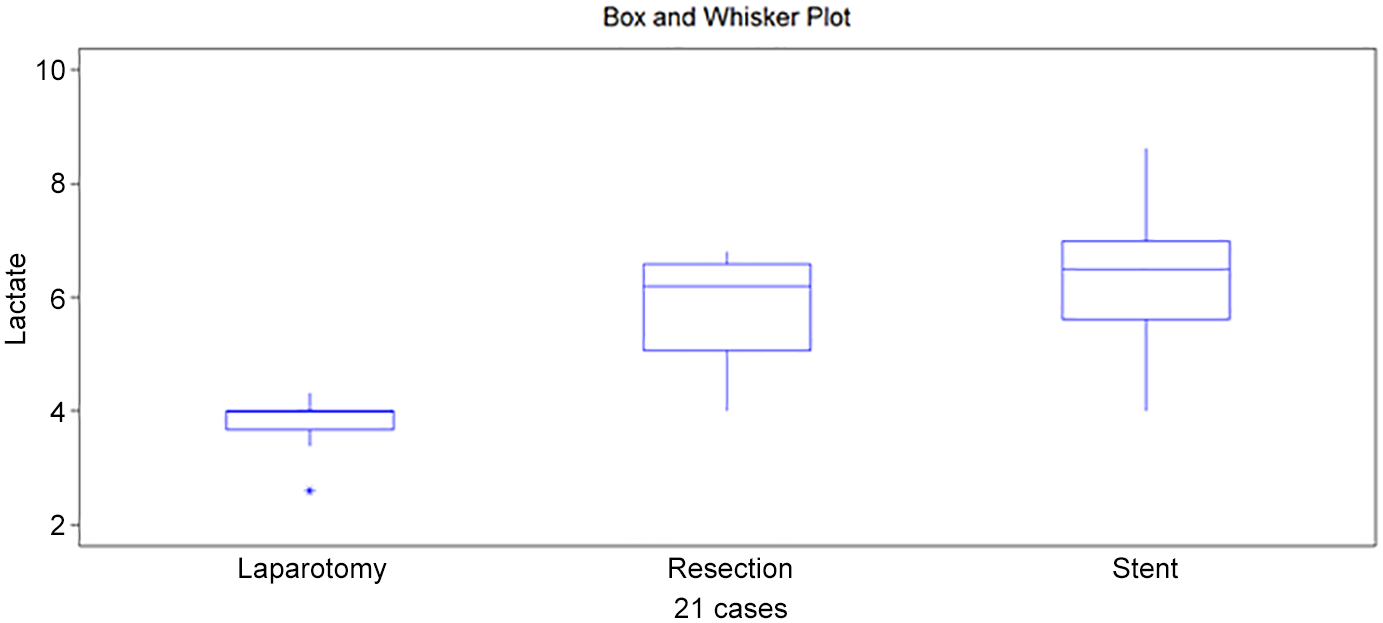
Box and whisker plot from lactate at colon 1.

**Figure 2:**
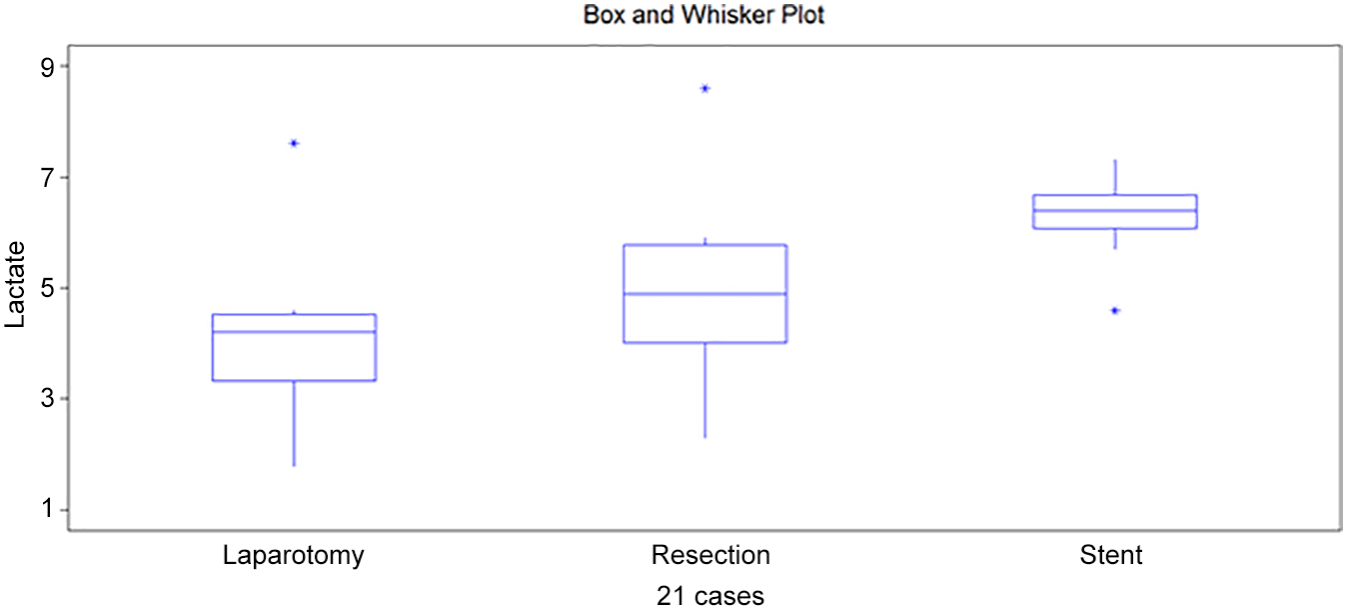
Box and whisker plot from lactate at colon 2.

**Figure 3:**
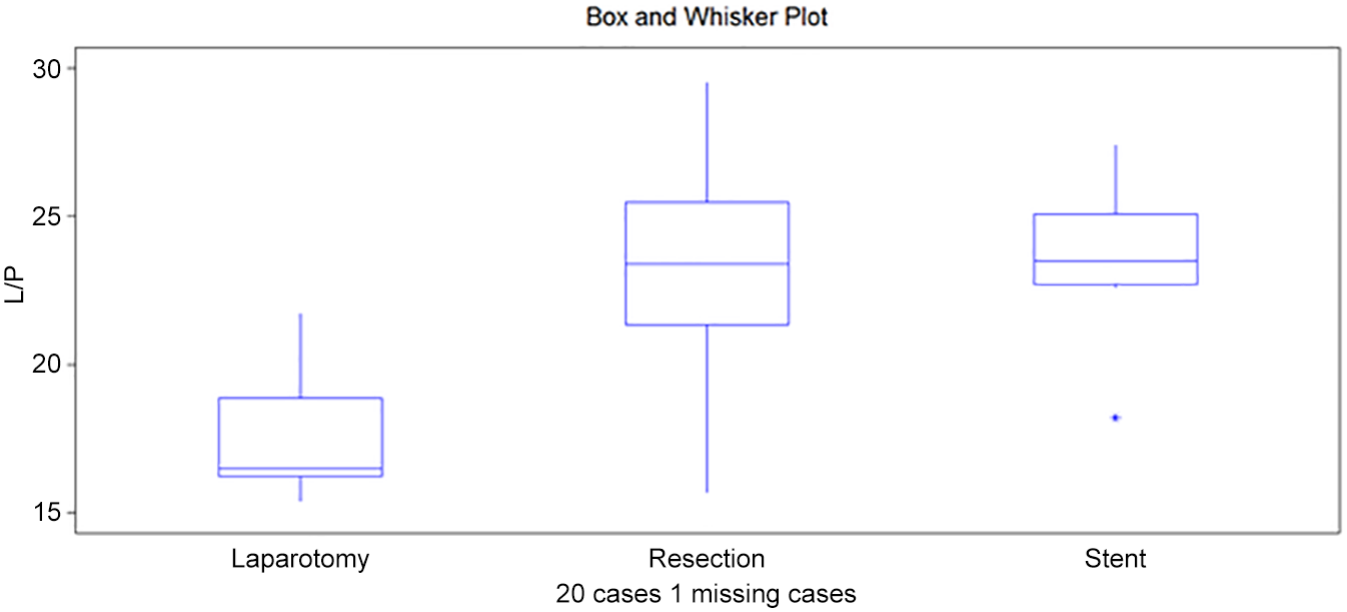
Box and whisker plot from L/P ratio at colon 1.

**Figure 4:**
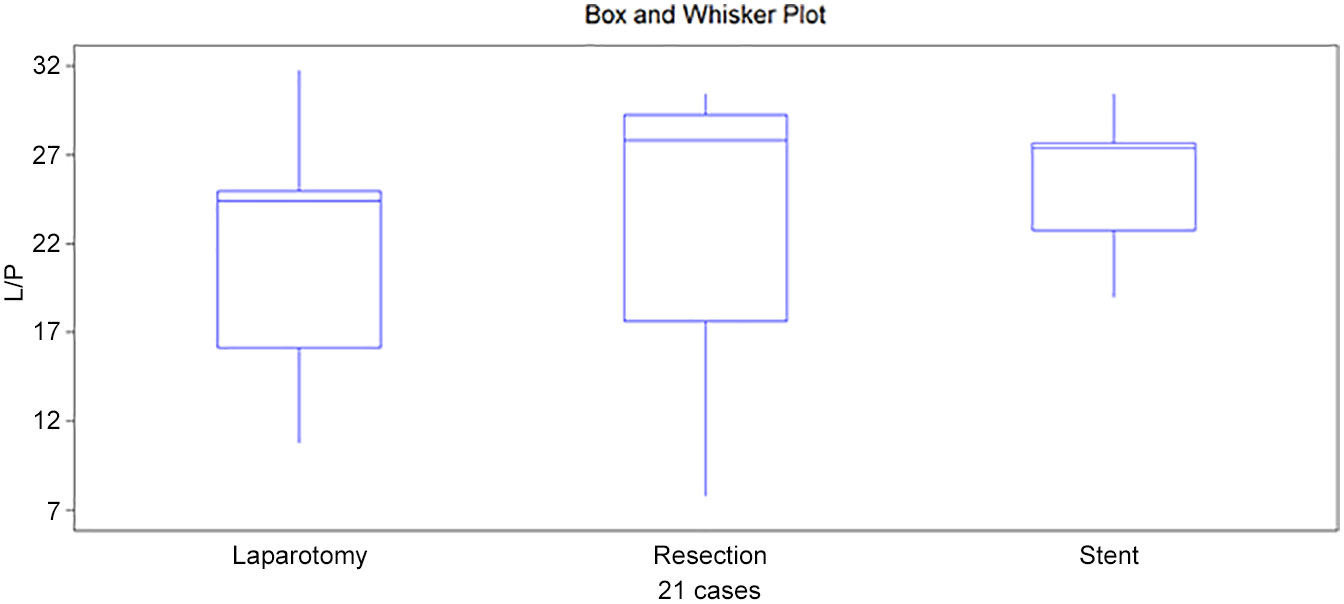
Box and whisker plot from L/P ratio at colon 2.

Fully covered self‐expandable metal stents (FCSEMSs) could be an option in the early treatment of AL in colorectal surgery if can be shown to be non‐detrimental in anastomotic healing by, for example, causing ischemia at the anastomosis. The aim of this pilot animal study was to investigate the local metabolic changes during the insertion of a fully covered self‐expandable stent under a colon anastomosis (Figs 1, 2, 3, 4).

## Methods

### Animals

In this study, we used seven healthy 3‐month‐old domestic pigs of both sexes (a cross‐breed between a Swedish country breed and Hampshire and Yorkshire breeds), with a mean body weight of 30.4 kg (26–34). The pigs were housed at room temperature at a farm, with free access to standard porcine fodder, before the experiment. They were kept in a 16‐h day and 8‐h night cycle. The experiment was approved by the Regional Animal Ethics committee in Linköping (ID 835‐Dnr 17869‐2020). The study was conducted in accordance with the guidelines of the European Union for the protection of animals used for scientific purposes. The animal experimentation in this study is reported according to the ARRIVE guidelines.^30^

### Anesthesia, fluid administration, ventilation, and euthanasia

Anesthesia was induced by tiletamine (6 mg/kg, i.m.; Virbac, Kolding, Denmark), zolazepam (6 mg/kg, i.m.; Virbac), and azaperone i.m. (4 mg/kg). In addition, atropine (1.5 mg, i.m.; Mylan, Stockholm, Sweden) was given. Propofol (1–2 mg/kg, i.v.; Fresenius Kabi, Uppsala, Sweden) was given if needed. Two peripheral catheters were inserted into the auricular veins of the experimental animals (1.1 mm, Venflon Pro Safety, BD, Helsingborg, Sweden). The animals were orally intubated in the prone position with a 6‐mm endotracheal tube (Covidien, Tullamore, Ireland). Anesthesia was maintained. The depth of anesthesia was intermittently (every 5 min) monitored by pain response at the cleft of the back leg. No muscle relaxants were given. Ringer acetate (10 mL/kg/h, i.v.; Fresenius Kabi) and 10% glucose with 40 mM sodium and 20 mM potassium (0.5 mL/kg/h, i.v.; Fresenius Kabi) were administrated by volume pumps (Alaris GP, CareFusion) to substitute for fluid loss. The pigs were ventilated using volume‐control ventilation (PV 501, Breas Medical AB, Sweden) to achieve arterial pCo_2_ of 5.0–5.3 kPa, and FiO_2_ was adjusted to maintain arterial PO_2_ at 12–18 kPa. The animals were covered with a thermal mattress; a forced‐air warming blanket was also used. At the end of the experiment, euthanasia was performed with a rapid i.v. injection of 40 mmol potassium chloride (B. Braun, Danderyd, Sweden), and asystole and circulatory arrest were confirmed with electrocardiogram and blood pressure recordings.

### Surgical preparation and measurements

A 4‐Fr introducer was placed in the right carotid artery by the open cut‐down method for the measurement of systemic blood pressure as well as blood sampling. The blood samples were analyzed for blood gases. A midline abdominal incision was performed. A 14‐Fr Foley catheter was inserted in the urinary bladder and fixed with purse‐string suture. Three microdialysis catheters were placed intraperitoneally (M‐dialysis gastrointestinal catheter 62). The first microdialysis catheter was sutured at the superior aspect of the descending colon, and the second microdialysis catheter was sutured in the colon 20 cm distal from the first one. A third reference catheter was placed and sutured at the small intestine. After the laparotomy, microdialysis measurements were performed. In the second part of the experiment, a resection of the colon between the first and second colon catheters close to the intestine was performed without disturbing its vascularization. Microdialysis samples were collected. Finally, at the third part of the experiment, an anastomosis between the colon stumps was constructed with a circular staple of 29 mm, and a fully covered, self‐expandable metal esophagus stent (Hanaro; NES‐28‐110‐070; Olympus) was placed over the anastomosis. Three syringe pumps (M‐dialysis106 pumps) were used to propel the solution. The microdialysate was analyzed (M‐dialysis ISCUS) for glucose, glycerol, pyruvate, lactate, and glycerol, and L/P was then calculated. The midline incision was sutured at the end of each procedure with a continuous suture.

### Protocol

In a prospective observational study, seven animals were postoperatively compared pairwise in three phases. The animals were first operated with laparotomy, and three MD catheters and a urine catheter were placed. This was followed by a 1‐h intervention‐free period. Three hours later, in the second phase of the experiment, colon resection was performed. At the third phase, 2 h after the colon resection, an anastomosis was constructed and the stent was inserted. Blood pressure, pulse, and urine production were measured after each phase of the experiment, and at the same time, IP samples were collected and analyzed immediately. Blood samples were collected from the carotid artery and were analyzed for blood gases.

### Intraperitoneal microdialysis

IPM is a method that uses a double‐lumen catheter with a diameter of 0.9 mm placed between the free‐floating small intestine loops. In this study, microdialysis equipment from M‐dialysis AB Stockholm Sweden was used. At the end of this catheter was a semipermeable membrane. The membrane pores were of two different sizes: the gastrointestinal catheter of 20 kDa pores allowed lactate, pyruvate, glucose, and glycerol to filter through the membrane. A microdialysis pump provided a constant flow to the semipermeable membrane, which facilitated the equilibrium of the ringer dialysate with the extracellular tissue. This sample was collected in a small test tube called a microvial. The microvial was analyzed by a computer‐assisted spectrophotometer. The analysis was performed continuously with 20‐min intervals between sampling, which was needed for the microvial to be filled with an adequate amount of fluid. The analysis of lactate, pyruvate, glucose, and glycerol takes 7 min, which basically allows continuous monitoring of the balance between aerobic and anaerobic metabolism at the cellular level.

### Statistical analysis

In the statistical analysis, pairwise comparisons between groups were performed after the colon resection and after the anastomosis with the stent placement using the Kruskal–Wallis test and anova (Statistix 8). Data are presented as median and interquartile range. A *P*‐value of <0.05 was regarded as statistically significant.

## Results

### Vital parameters

Blood pressure was stable during the experiment with only small variations, with an average pressure of 80 mmHg (Table 1). Small variations throughout the experiment could also be noted in the pulse frequency, with approximately 90 beats/min. Urine production was stable throughout the experiment between 35 and 65 mL/h. No statistical differences could be seen in the vital parameters during the experiment.

**Table 1:** Vital parameters of average blood pressure (mmHg), pulse (beats/min), and urine production (mL/h) measured after laparotomy, resection, and anastomosis with stent insertion. (median values, Q1/Q3)

	Laparotomy	Resection	Stent	*P*‐value
Blood pressure	78	82	77	0.769
Q1/Q3	69/87	73/96	72/90	
Pulse	89	100	92	0.139
Q1/Q3	77/95	90/113	89/113	
Urine production	35	65	50	0.994
Q1/Q3	30/85	25/70	40/55	

### Arterial acid–base results

Completely stable arterial pH could be seen during the study (7.56), but a variation in base excess (BE) could be noted, with it increasing from 7.3 mmol/L after the laparotomy to 7.7 mmol/L after the colon resection (Table 2). A decline was seen after anastomosis and stent insertion, as BE decreased to 7.5 mmol/L. PO_2_ decreased during the experiment, starting at 16.3 kPa and declining to 15.1 after resection, with a final value of 14.3 mmol/L after stent insertion. PCO_2_ was stable at 4.5 KPa throughout the experiment.

**Table 2:** Arterial acid–base samples after laparotomy, colon resection, and anastomosis with stent insertion (median values, Q1/Q3)

	Laparotomy	Resection	Stent	*P*‐value
pH	7.56	7.55	7.56	0.708
Q1/Q3	7.55/7.58	7.5/7.59	7.5/7.6	
BE	7.3	7.7	7.5	0.772
Q1/Q3	6.6/8.2	6.8/8.4	6.7/10.1	
PO_2_	16.3	15.1	14.3	0.118
Q1/Q3	15.6/17.5	13.2/15.6	11.9/17.0	
PCO_2_	4.4	4.5	4.4	0.934
Q1/Q3	4.3/4.6	4.2/5.4	4.1/5.5	
Glucose	5.5	5.5	5.4	0.570
Q1/Q3	5.0/6.1	5.3/5.8	5.0/5.6	
Lactate	1.6	1.5	1.3	0.2248
Q1/Q3	1.3/1.8	1.2/2.3	0.9/1.8	

BE, base excess.

Arterial glucose was completely stable during the experiment with values of 5.5 and 5.4 mmol/L during the three observations. A gradually decreasing trend could be seen in arterial lactate, and after laparotomy, the median value of lactate was 1.6 mmol/L, which decreased to 1.5 mmol/L after resection and, finally, to 1.3 mmol/L after stent insertion.

### Microdialysis results

Glucose at colon 1 (proximal to colon resection) was 3.0 mmol/L after laparotomy, which decreased to 2.9 mmol/L after colon resection and further dropped to 2.5 mmol/L after stent insertion. This reduction was, however, not significant (*P* = 0.601) (Table 3).

**Table 3:** Microdialysis results at colon 1 (proximal to anastomosis), colon 2 (distal to anastomosis), and at the reference catheter at the middle of small intestine (median values, Q1/Q3)

	Laparotomy	Resection	Stent	*P*‐value
Glucose, colon 1	3.0	2.9	2.5	0.601
Q1/Q3	1.9/5.1	2.3/5.1	2.1/4.1	
Glucose, colon 2	2.9	4.3	2.2	0.070
Q1/Q3	2.4/4.2	3.4/6.9	1.2/4.5	
Glucose, small intestine	2.4	3.9	4.3	0.853
Q1/Q3	2.3/6.6	2.5/4.8	0.5/4.5	
Lactate, colon 1	4.0	6.2	6.5	0.0035
Q1/Q3	3.4/4.0	4.6/6.7	5.0/7.0	
Lactate, colon 2	4.2	4.9	6.4	0.0549
Q1/Q3	2.5/4.6	3.4/5.9	5.7/6.8	
Lactate, small intestine	4.0	5.8	6.0	0.161
Q1/Q3	2.9/6.3	4.5/7.6	4.7/6.6	
Pyrnvate, colon 1	216	232	226	0.486
Q1/Q3	167/262	186/290	211/357	
Pyrnvate, colon 2	185	254	233	0.404
Q1/Q3	174/239	169/284	211/241	
Pyrnvate, small intestine	180	207	260	0.157
Q1/Q3	72/184	97/286	244/290	
Glycerol, colon 1	61	49	52	0.400
Q1/Q3	40/95	20/62	44/59	
Glycerol, colon 2	51	51	54	0.756
Q1/Q3	42/90	37/65	44/69	
Glycerol, small intestine	78	37	37	0.140
Q1/Q3	32/143	17/75	26/47	
L/P ratio, colon 1	16.5	24.2	23.5	0.0144
Q1/Q3	15./19.4	21.3/29.5	22.6/26.5	
L/P ratio, colon 2	24.4	27.8	27.4	0.590
Q1/Q3	15.7/25.5	11.9/29.5	20.9/28.0	
L/P ratio, small intestine	23.1	28.1	22.6	0.634
Q1/Q3	19.8/35.0	21.3/78.2	19.7/27.0	

Glucose at colon 2 (distal colon) started after the laparotomy similar to glucose at colon 1 at 2.95 mmol/L but then increased after the resection to 4.3 mmol/L, after which it almost halved to 2.2 mmol/L after the stent placement. Changes were, however, not significant (*P* = 0.070).

Glucose at the small intestine (reference catheter) after laparotomy was 2.4 mmol/L, which gradually increased to 3.9 mmol/L after resection and further to 4.3 mmol/L after stent insertion (*P* = 0.853).

Lactate at colon 1 after laparotomy was 4.0 mmol/L, which then increased to 6.2 and 6.5 mmol/L after resection and anastomosis with stent insertion, respectively (*P* = 0.035) (Fig. 1).

Median value of lactate at colon 2 was 4.2 mmol/L after laparotomy, which then increased to 4.9 mmol/L after colon resection and finally reached 6.4 mmol/L after anastomosis and stent placement. This increase was not significant (Kruskal–Wallis test; *P* = 0.0549). However, with anova the *P*‐value was 0.046 (Fig. 2).

Reference lactate at the small bowel started at 4.0 mmol/L after laparotomy and increased to 5.8 mmol/L after resection and further to 6.0 mmol/L after stent placement (*P* = 0.161).

Pyruvate at colon 1 showed small differences in data values with 216, 232, and 226 μmol/L, respectively, at the three measurement occasions. At colon 2, a slightly greater variation could be seen 185, 254, and 233 μmol/L, respectively. At the small intestine, the corresponding values were 180, 207, and 260 μmol/L, respectively.

Glycerol at colon 1 was 61 μmol/L after laparotomy, which dropped after resection to 49 μmol/L, with a slight recovery to 52 μmol/L after stent placement. At colon 2, stable values could be seen at 51, 51, and 54 μmol/L, respectively. In the reference catheter of the small intestine, an initial value of 78 μmol/L after the laparotomy was noted, which declined sharply to 37 μmol/L after resection and stent insertion (*P* = 0.400 and 0.756, respectively).

An L/P value of 16.5 was obtained after laparotomy, which increased to 24.2 after resection; thereafter, a slight decrease to 23.5 was noted after stent insertion. The differences were significant (*P* = 0.014) (Fig. 3).

A similar pattern was seen in L/P at colon 2. An initial value of 24.4 increased after resection to 27.8 and decreased slightly after anastomosis and stent placement to 27.4. Large variations in values at the different locations had, however, no statistical significance (*P* = 0.590) (Fig. 4).

The reference catheter located in the small intestine showed a similar pattern as in colon 1 and colon 2, with an initial value of 23.1, which increased to 28.1 after the resection but decreased to 22.6 after stent insertion (*P* = 0.634).

## Discussion

An intraluminal covering stent has been shown to be a successful treatment for esophageal rupture in AL after gastric bypass or esophageal cancer surgery.^6^, ^7^, ^8^ Covering colon stents have been tested in AL after colon surgery, and the preliminary results seem promising.^9^, ^10^, ^11^, ^12^ A leading cause of AL in colorectal surgery is ischemia in the proximal and/or distal intestine at the anastomosis, which gives rise to leakage several days after the operation. The purpose of this study was to observe how an intraluminal stent affects the intestinal metabolism in the intestinal wall proximally and distally of the stapled colon anastomosis over a colon stent.

Our study results show that the vital parameters as well as the acid–base samples were stable during the experiment and showed no differences after laparotomy, resection, or anastomosis/stent insertion. In the microdialysis response, several changes were found at the different measurement locations.

Lactate increased significantly proximal to resection (colon 1) and distal to resection (colon 2), while at the reference catheter (small intestine) only a slight but non‐significant increase could be noted.

The ratio L/P increased significantly at colon 1, while a high level of this ratio after laparotomy was noted at colon 2. At both locations, the values further increased after the resection, while a slight decrease occurred after anastomosis and stent insertion. No significant differences at the small intestine could be detected.

Glucose measurements showed a slight decrease at colon 1 after anastomosis with stent insertion, while those at colon 2 decreased more but not significantly after anastomosis and stent insertion. These values showed a pattern similar to that of L/P, which increased after resection and then decreased after anastomosis and stent insertion.

Pyruvate values showed a tendency similar to that of glucose, with the highest values seen after resection. Glycerol value decreased at the small intestine during the course of the experiment, but it basically remained unchanged both proximally and distally to the anastomosis.

In the carbohydrate metabolism of the cell, glucose is transported into the cell in presence of insulin, where it is converted into pyruvate if oxygen is available; the reactions proceed through coenzyme A into the Krebs cycle, and a large amount of energy in the form of Adenosine triphospate (ATP) is recovered. In an anaerobic situation, pyruvate is converted to lactate, and significantly less energy is recovered. L/P reflects the current relationship between aerobic and anaerobic metabolism.^31^ In an ischemic situation, L/P is high, lactate is high, and the glucose level approaches zero values. In a situation of hypermetabolism, glucose, lactate, and L/P increase.^5^

In colon 1 (proximal to resection and anastomosis) and colon 2 (distal to resection and anastomosis), our study shows a gradual increase in lactate from laparotomy to resection; it increase further after the third phase of the experiment, with anastomosis and stent placement. The ratio L/P increases from laparotomy to the resection stage, after which a minor decrease is seen after anastomosis and stent insertion. The same pattern as the L/P assays is seen in the glucose assays: glucose increases from laparotomy to the resection phase and then decreases. At the reference catheter (small intestine), glucose, pyruvate, and lactate increase successively, while L/P increases after resection and decreases slightly after anastomosis and stent insertion.

The changes in this study can be interpreted as due to hypermetabolism occurring both proximally and distally in the intestinal ends after colon resection (even though the commencement of a hypoxic reaction distal to the anastomosis (colon 2) cannot be fully ruled out). Hypermetabolism neither deteriorated nor turned into ischemia after anastomosis and stent insertion.^28^

In this study, anastomosis was carried out with a 25‐mm circular stapler, and the width of the stent was 29 mm. Thus the stent has 14% more width than the anastomosis, which means that the intestine next to the anastomosis is stretched, thus increasing risk of ischemia and necrosis under the stent; however, we did not notice such signs during the study period. The stapled anastomosis together with the stent insertion could reflect the hypermetabolism seen in the study.

The study has several limitations. Only a few experimental animals were used, and the study time was limited to 9 h, which may be considered short, as most anastomotic leaks are diagnosed much later in the postoperative course. Therefore, an extended study to evaluate the effects of the stent insertion over a stapled colon anastomosis is recommended. Nevertheless, we conclude that colon resection causes hypermetabolism in the intestinal ends next to the resection site and at the small intestine and that hypermetabolism neither deteriorates nor turns into ischemia during the initial postoperative course when a colon stent is placed intraluminally under a stapled anastomosis. However, an initial start of hypoxia most pronounced at the distal end of the colon resection cannot be excluded.
